# Intra-molecular lysine-arginine derived advanced glycation end-product cross-linking in Type I collagen: A molecular dynamics simulation study

**DOI:** 10.1016/j.bpc.2016.09.003

**Published:** 2016-11

**Authors:** Thomas A. Collier, Anthony Nash, Helen L. Birch, Nora H. de Leeuw

**Affiliations:** a Department of Chemistry, University College London, 20 Gordon Street, London WC1H 0AJ, United Kingdom; b Institute of Orthopaedics and Musculoskeletal Science, UCL, RNOH Stanmore Campus, London, United Kingdom; c School of Chemistry, Cardiff University, Cardiff CF10 1DF, United Kingdom

**Keywords:** Collagen, Glycation, Molecular dynamics, Protein cross-linking, Glucosepane, Advanced glycation end products, DOGDIC

## Abstract

Covalently cross-linked advanced glycation end products (AGE) are among the major post-translational modifications to proteins as a result of non-enzymatic glycation. The formation of AGEs has been shown to have adverse effects on the properties of the collagenous tissue; they are even linked to a number of age related disorders. Little is known about the sites at which these AGEs form or why certain sites within the collagen are energetically more favourable than others. In this study we have used a proven fully atomistic molecular dynamics approach to identify six sites where the formation of the intra-molecular 3-deoxyglucosone-derived imidazolium cross-link (DOGDIC) is energetically favourable. We have also conducted a comparison of these positions with those of the more abundant glucosepane cross-link, to determine any site preference. We show that when we consider both lysine and arginine AGEs, they exhibit a prevalence to form within the gap region of the collagen fibril.

## 1 Introduction

Collagen is an essential protein in the extracellular matrix (ECM) that is responsible for an important number of structural and functional processes within connective tissues [1]. The different collagen types form a diverse family of proteins accounting for over a quarter of the dry mass of the human body [2].

Fibril-forming type I collagen is the most abundant collagen type and is found in organs and tissues that require tensile strength, such as tendon, ligament, bone, and the dermis. Type I collagen molecules are composed of two α1 and one α2 polypeptide chains twisted into a continuous triple helix, flanked on both ends by non-helical telopeptides. Collagen molecules have a length of about 300 nm and a triple helical diameter of 1.5 nm. Once secreted by the cell into the ECM they tightly self-assemble to form micro-fibrils in a process called fibrillogenesis, which according to the Hodge-Pertruska model sees the collagen molecules aligned both parallel and staggered with respect to one another [3]. By aligning in this way an observable periodicity is created, known as the D-band, where D = 67 nm. The D-band can be subdivided into two further regions; the gap-region (0.54D) between two aligned consecutive collagen molecules and a period of higher protein density known as the overlap region (0.46D). Microfibrils further associate laterally to form fibrils, which, depending on the organism and tissue, can have diameters of between 20 and 500 nm and a length in the millimetre scale [4], [5]. Enzyme-mediated cross-links form between the telopeptide regions of collagen molecules within a fibril to give the ordered bundle of collagen molecules high tensile strength [6]. It is not only this strength that makes collagen an important component of the ECM, but also the large number of biomolecule binding events that collagen is involved in [7].

During chronological ageing the properties of collagen slowly change, which is thought to be due mainly to glycation and the subsequent formation of advanced glycation end-product (AGE) cross-links within and between collagen molecules. AGEs are formed by a series of successive chemical reactions between a reducing sugar, such as glucose (an aldose) or fructose (a ketose), and a protein or lipid. Unlike enzymatic cross-links, which infer functionality to tissues, AGEs are pathological and alter the performance of collagenous tissues. Type I collagen is particularly prone to AGE cross-linking in a number of different tissues, owing to its long half-life, which can be up to 200 years in tendon [8]. Studies have shown that the mechanical and biological functions of collagen are disrupted or altered upon formation of AGE cross-links. Reigle et al. showed weaker cell adhesion to a glycated collagen matrix, which they attributed to a reduced binding affinity by the collagen proteoglycan [9]. Reddy et al. found that in vitro incubation of rabbit Achilles tendon in ribose increased levels of the AGE pentosidine and they also observed an increase in the Young's modulus by 159% from 24.89 ± 1.52 MPa to 65.087 ± 14.41 MPa, suggesting that the presence of AGE cross-links increases the stiffness of soft tissue [10].

**Fig. 1:**
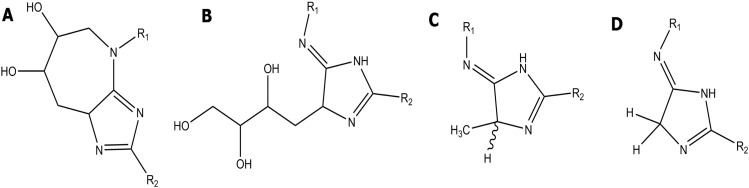
Schematic image of Lysine (*R*_1_)-Arginine (R_2_) cross-linking AGEs, A) Glucosepane B) DOGDIC, C) MODIC and D) GODIC.

So far, only a few physiologically relevant AGEs have been characterised from tissues ex vivo, notably lysine-lysine and lysine-arginine cross-link forming AGEs. There are four main lysine-arginine AGE cross-links (Fig. 1): glucosepane, DOGDIC, MODIC and GODIC, which differ in the reactive dicarbonyl donors, i.e. glucose, deoxyglucosone, methylglyoxal and glyoxal, respectively. A 2002 study by Biemel et al. has quantified the levels of these AGE cross-links in human lens protein and found concentrations of 132.3–241.7 pmol/mg of protein for glucosepane, 1.3–8.0 pmol/mg of protein for DOGDIC, 40.7–97.2 pmol/mg of protein for MODIC and concentrations below the quantifiable level of the instrument for GODIC [11]. Differences in the levels of these AGE cross-links may result from a variation in the concentrations of attacking carbonyl substrates in the tissue, or from a difference in the energetic favourability of the particular AGE cross-link. It is not known which is the dominant factor responsible for determining the levels during the ageing process in the body.

In this study, we identify energetically favourable formation sites for DOGDIC cross-link formation and probe the subsequent structural changes of the collagen. In previous work, we have identified sites of glucosepane formation within the collagen molecule, using an all-atom Molecular Dynamics (MD) approach, and discussed the effect that their location may have on the biological function of the collagen. In this study we focus on DOGDIC, which allows a direct comparison with the much more prevalent glucosepane, as both cross-links form between lysine and arginine residues, using d-glucose via the Schiff base, unlike MODIC and GODIC which form via glucose degradation products methylglyoxal and glyoxal. In view of the very large difference in concentrations between the two AGEs, we aim to determine whether there is a difference in the number of energetically favourable formation sites for glucosepane and for DOGDIC and whether there is an overlap between favourable sites of the cross-link formations within the collagen molecule.

## 2 Theoretical methods

### 2.1 Constructing the model

The collagen I model used has been fully described in previous works [12], [13], where the sequence and structural information is for the *Rattus norvegicus* organism. The sequence information is detailed in the UniProt entries COL1A1 (P02454) and COL1A2 (P02466) [14], including the post-translational modified residues such as hydroxyproline and hydroxylysine, present in the UniProt entries. The structural basis for the model came from the crystal structure derived Protein Data Bank entry 3HR2 [15]. The linear telopeptides and side chain atoms were finally added using LeaP, part of the AMBER12 software package [16]. The system is run at physiological pH, which results in a net charge of + 33, and to neutralise this positive charge, 33 chloride ions are added to the system. Finally 11,980 explicit water molecules are added to the system using LeaP, which equates to a fibrillar water content of 0.75 g water g^− 1^ of collagen, which is in good agreement with experiment [17].

A single DOGDIC cross-link was inserted into the collagen molecule between the residues identified during the distance-based criterion search (see below), totalling 24 unique models of collagen molecules with a single cross-link. A reference system of a native collagen molecule without cross-links but with an unbound d-glucose and three fewer water molecules is also generated to allow the calculation and direct comparison of relative thermodynamics.

### 2.2 Distance-based criterion search

A custom script was used to scan the triple helical portion of a low energy conformer of the collagen molecule to identify any lysine and arginine residues within a 5 Å cut-off across separate polypeptide chains. The 5 Å distance used for the search was chosen, as it is double the distance of the shortest separation between the nitrogen atoms within DOGDIC (approximately 2.5 Å and 3.5 Å) [18]. The distance was calculated between the three terminal nitrogen atoms of lysine N^ζ^ and arginine N^η^, the lysine C^ε^ and the arginine N^ε^, and lysine C^δ^ and arginine C^δ^. Any site where at least one distance criterion was met was considered for cross-linking.

### 2.3 Molecular dynamics simulations

MD simulations were performed using SANDER, part of the AMBER12 software package [16], exploiting periodic boundary conditions to replicate the densely packed fibrillar environment. Simulations are run using the ff99SB force field, with additional terms for the non-standard amino acids, hydroxyproline and hydroxylysine. Covalent bond lengths involving hydrogen were restrained using the SHAKE algorithm, such that a time step of 2 fs could be adopted [19], whereas the non-bonded interactions are described by a pairwise additive Lennard-Jones 6–12 potentials and pairwise additive coulombic potentials. Coulombic potentials were calculated using the Particle Mesh Ewald summation with a cut-off radius of 8.0 Å. Constant temperature and pressure were maintained with the Berendsen algorithm [20], using a barostat time constant of 5.0 ps atm^− 1^ and a thermostat time constant of 1.0 ps. Anisotropic coordinate rescaling rather than isotropic rescaling for maintaining the constant pressure is employed owing to the long-thin nature of the unit cell.

The initial model undergoes steepest descent energy minimisation followed by conjugate gradient minimisation to reduce the initial instabilities within the model. The system was then heated to 310 K for 120 ps using the NVT ensemble followed by a further 320 ps using the NPT ensemble. Production simulations ran for 60 ns at 310 K using the NPT ensemble. Analysis was performed over the final 25 ns of the production simulations, as the system density and the potential energy converged at 35 ns.

## 3 Results

Adopting a similar approach as in our previous study [13], we have identified sites of preferential DOGDIC formation. First, a distance-based criterion search was used across a collagen type I molecule to identify lysine and arginine residues within 5 Å of one another. Next, a number of periodic atomistic MD simulations were performed under physiological conditions of a collagen molecule with a single intra-molecular DOGDIC cross-link for each of the identified lysine-arginine sites. The use of periodic boundary conditions exploiting the D-band periodicity enables the replication of the dense fibrillar environment of the collagen matrix. A site is a likely candidate for DOGDIC cross-link formation if the energy of the singularly cross-linked collagen molecule is less than the energy of the reference model; a native collagen molecule in the presence of an open-chain glucose molecule minus three water molecules. The potential cross-linking sites are then transposed onto the candidate cell and matrix interaction domain map of the collagen molecule produced by Sweeney et al. [21], in order to identify the possible impact of the DOGDIC cross-link on biomolecular processes in the ECM.

**Fig. 2:**
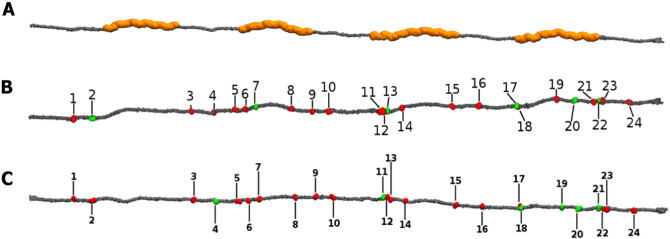
Shows a collagen molecule with A. gap regions highlighted in orange, B. the favourable (green) and unfavourable (red) potential glucosepane cross-linking sites C. the favourable (green) and unfavourable (red) potential DOGDIC cross-linking sites.

The distance-based criterion search along the length of a collagen molecule simulated in its native state for 100 ns, identified 24 sites within a 5 Å cut-off. The distribution of these sites over the length of the collagen molecule is shown in Fig. 2. It is immediately apparent that a larger number of favourable cross-linking sites are located within the gap region of the collagen molecule.

The DOGDIC and reference model simulations were performed for 60 ns using the NPT ensemble. The total energies from the final 25 ns were used to calculate average binding enthalpies, as it was shown that at 35 ns the system density and potential energy had converged. Using this approach, six sites were identified where DOGDIC formation was energetically favoured, with the binding enthalpies for all sites given in Table 1. The standard error of the mean was calculated to determine the statistical error in the formation enthalpy for all of the simulations, which was found to be approximately 0.7 kcal mol^–1^.

The energetically favoured sites were mapped onto the candidate cell and matrix interaction domain map, revealing a number of overlaps with regions of biological significance (See Table 2). Site 4 occurs at the interaction sites of α2β1 integrin, IL-2 and an HSP-47 chaperone [22], [23], [24]. Site 11 is local to the binding sites of HSP-47 chaperone, phosphoryn and the proteoglycan keratan sulfate (KS) [21], [22], [25], [27]. Site 18 is within the binding sites of α2β1 integrin, a HSP-47 chaperone, which is a fibrillogenesis inhibitor, and is also within close proximity of the binding site of the collagenase MMP-1 [22], [24], [26]. Site 19 occurs within the binding site for IL-2, HSP-47 chaperone and the proteoglycan DS [22], [23], [27]. Site 20 is within the binding site of DS proteoglycan and IL-2 [23], [27]. Finally, site 21 is within the IL-2 binding domain, as well as the binding location of the HSP-47 chaperone [22], [23].

The number of identified potential sites for DOGDIC formation equaled those identified for glucosepane formation, yet only one site (site D20) overlapped, although at this site glucosepane formation is more exothermic.

## 4 Discussion

Our study revealed potential sites within the collagen molecule where DOGDIC formation is favourable. The distance-based criterion search along the length of the collagen molecule found 24 sites, where the separation of the lysine and arginine residues on different polypeptide chains was below 5 Å. Of those 24 identified sites, only 6 were found to have an exothermic change in enthalpy upon formation of the DOGDIC cross-link, when compared to that of the reference model. At the favourable DOGDIC cross-linking sites no significant deviation was observed in the position of the backbone atoms. However, rotation of the side-chains around the alpha-carbon atom was observed. It can be inferred that, like glucosepane, the major contribution to the decrease in enthalpy is an increase in the number of non-bonded interactions upon cross-link formation and rearrangement of the local environment. The chloride ions, added to balance the positive charge of the cationic amino acid residues, were found evenly distributed throughout each model, at similar positions in both the native and cross-linked models. As the closest proximity of a DOGDIC cross-link to chloride ions falls outside of the electrostatic cut-off (8 Å), the presence of chloride ions will not influence the energetics of cross-link formation in our simulations.

**Fig. 3:**
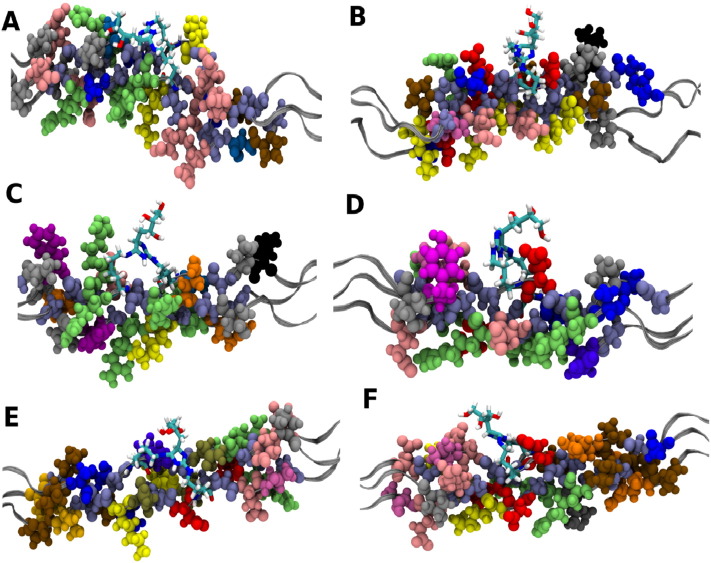
Local environment around the favourable DOGDIC cross-link sites a) Position 4, b) Position 11, c) Position 18, d) Position 19, e) Position 20 and f) Position 21. (Residue colours: Ala — blue; Asn — tan; Asp — red; Arg — lime; Gln — orange; Glu — pink; Gly — ice blue; His — violet; Hyp — silver; Ile — grey; Leu — black; Lys — yellow; Met — white; Phe — purple; Pro — ochre; Ser — light blue; Thr — mauve; Tyr — magenta; Val — gold); glucosepane cross-link shown as sticks.

The six favourable cross-link sites are presented in Fig. 3, along with their neighbouring amino acids. Noticeable non-bonded interactions are formed during the simulations, for example between a backbone carboxyl group and a sidechain of the cross-linked residue at position 11, where a hydrogen-bonding interaction between ^734^Arg (α1a) HE and ^733^Gly (α1a) O with an average bond length of 2.07 Å is formed in the last 25 ns of the simulation. There are also side-chain to side-chain interactions, for example a potential hydrogen-bonding interaction at site 21, between ^1084^Asp (α1a) OD2 and ^1082^Arg (α1b) HH2, with an average bond distance of 1.75 Å.

The interactions observed for DOGDIC differ slightly from those observed for glucosepane in two ways. Firstly, there are non-bonded interactions at the DOGDIC sites, that are not observed at the sites for glucosepane and which occur between the side-chains of two cross-linking residues, for example at site 21 where we see a potential hydrogen-bond between ^1085^Lys (α1a) O and ^1082^Arg (α1b), with an average separation of 2.16 Å. Secondly, there are noticeably fewer cross-link to side-chain or cross-link to backbone interactions. This can potentially be explained by two major differences between the two different cross-links; the first is the greater flexibility of the DOGDIC cross-link, in particular the four carbon aliphatic chain, and the second is the greater polarity of the DOGDIC cross-link itself, which means that it is more likely to be involved in water-mediated hydrogen-bonding, if not in direct hydrogen-bonding.

A comparison between the number of favourable DOGDIC and glucosepane formation sites [13], reveals an equal propensity for each AGE cross-link formation, although of the six DOGDIC sites, only site 20 was identified in both studies. The most likely reason for this variation is due to the difference in the separation between the three terminal nitrogen atoms lysine N^ζ^ and arginine N^η^. In glucosepane these separations are 2.6 Å and 3.8 Å, respectively, whereas in DOGDIC the separation is smaller at 2.5 Å and 3.5 Å, which is potentially the determining factor as to whether cross-link formation will be favourable. Upon rearrangement of the side-chains the resulting configuration may impose close contacts of those side-chains with their neighbouring residues. The difference between the two sets of distances suggests that the structure of the residues at the same site for DOGDIC and glucosepane is not the same. In one structure an unfavourable close contact may be introduced or a favourable electrostatic interaction may be missing, depending on the cross-link formed. Another possible explanation is the difference in the degree of polarity between the two cross-links, with glucosepane having two hydroxyl groups whilst DOGDIC has three. However, there is no net change in the polarity, leading us to believe that this is unlikely to be a significant contributor. The one common site, site 20, is seen in Fig. 3 to have fewer bulky amino acid residues surrounding the cross-link. This means that it is likely to have a greater flexibility in its movement and thus the ability to form the cross-link without the introduction of close-contacts or unfavourable interactions with neighbouring residues. It is, therefore, found to be energetically favourable for both of the studied AGEs.

Despite there being an equal number of favourable glucosepane and DOGDIC sites within the collagen molecule, other factors may affect the formation within the tissue; accounting for the difference in the reported ex vivo concentrations. For example, it has previously been reported that the dehydration step in the glucosepane formation is non-reversible, whereas DOGDIC formation is reportedly reversible, thus, potentially accounting for the difference in relative abundance in ex vivo [11]. Moreover, the simulations conducted in this study do not take into account activation barriers for cross-link formation, nor do they take into account the kinetics of the reaction; QM/MM approaches would allow activation barriers to be studied, but these are beyond current computational capabilities for the size of the system.

The potential impact of cross-link formation at the matrix-biomolecule binding sites has been discussed previously [13]. However, the location of the cross-links within the collagen molecule (Fig. 2) is interesting. What is immediately apparent is that all but one of the DOGDIC cross-links are located within the gap region of the collagen fibril. Glucosepane shows a similar affinity for cross-link formation in the gap regions of the collagen. There are two potential explanations for this observation. First, both AGEs are polar and hence capable of forming hydrogen-bonds to the intra-fibrillar water molecules; the number of water molecules per unit volume in the fibril is 20% higher in the gap region than in the overlap region [12], [29]. Second, the overlap region has a higher protein density (volume), which results in an increased likelihood of an unfavourable interactions occurring between the newly formed AGEs and the neighbouring collagen molecules. The influence of both of these factors can be seen in the conformations of the DOGDIC cross-link shown in Fig. 3. The one cross-link not located in the gap region shows a configuration with the hydroxyl chain of the DOGDIC cross-link running flat (parallel) to the backbone, whereas those in the gap region have the DOGDIC hydroxyl-chains perpendicular to the backbone, thus maximizing the amount of hydrogen-bonds to the intra-fibrillar water molecules.

In conclusion, we have identified six sites where the intra-molecular formation of DOGDIC cross-links in type I collagen is energetically favourable. We have then shown that the reduced N—N intra-distance in DOGDIC means that there is little competition for lysine-arginine sites with glucosepane, as they form exothermically at different sites. Our results suggest that lower levels of DOGDIC in human lens tissue is most likely the result from differences in the availability of carbonyl metabolite, or the non-reversibility of the glucosepane formation mechanism. Our final observation is that the two AGEs studied show a preference to form energetically in the gap region, owing to the lower protein density and higher intra-fibrillar water content.

## Figures and Tables

**Table 1:** The enthalpy of formation of all 24 identified intra-molecular DOGDIC cross-links. Column 1 gives the site number, columns two to four highlight the cross-linked residue pair between two of the three polypeptide chains (labelled using the UniProt residue number and the triple helical residue number shown in brackets). The fifth column lists the change in enthalpy (kcal/mol), upon DOGDIC cross-link formation and the sixth column contains the enthalpy change upon glucosepane formation, taken from Collier et al. [13].

Cross-link	Chain α1 (a)	Chain α1 (b)	Chain α2	DOGDIC Δenthalpy	Glucosepane Δenthalpy (Collier et al.)
D1	^229^ARG(62)	^226^LYS(59)		+ 85.78	–
D2		^257^ARG(90)	^183^LYS(87)	+ 109.66	–13.57
D3		^419^LYS(252)	^348^ARG(252)	+ 50.08	+ 38.54
D4	^458^ARG(291)		^386^LYS(290)	–8.68	+ 7.88
D5		^494^LYS(327)	^419^ARG(323)	+ 20.21	+ 39.18
D6	^509^LYS(342)		^438^ARG(342)	+ 25.38	+ 4.36
D7	^527^LYS(360)		^456^ARG(360)	+ 11.61	–2.30
D8	^587^ARG(420)		^516^LYS(420)	+ 72.09	+ 43.33
D9	^620^ARG(453)		^549^LYS(453)	+ 55.07	+ 76.64
D10		^646^LYS(479)	^579^ARG(483)	+ 58.68	+ 4.08
D11	^734^ARG(567)	^731^LYS(564)		–14.33	+ 23.16
D12	^740^LYS(573)		^669^ARG(573)	+ 1.03	+ 19.28
D13	^748^LYS(581)		^677^ARG(581)	+ 9.03	–23.97
D14		^770^LYS(603)	^699^ARG(603)	+ 30.74	+ 73.65
D15	^854^ARG(687)	^851^LYS(684)		+ 83.03	+ 92.73
D16	^896^LYS(729)		^825^ARG(729)	+ 23.38	+ 55.40
D17	^958^LYS(791)	^956^ARG(789)		+ 53.69	–2.32
D18	^958^LYS(791)		^884^ARG(788)	–20.38	+ 65.52
D19	^1025^ARG(858)	^1022^LYS(855)		–61.58	+ 16.13
D20	^1055^ARG(888)		^980^LYS(884)	–4.85	–34.50
D21	^1085^LYS(918)	^1082^ARG(915)		–1.62	+ 21.91
D22	^1094^ARG(927)		^1020^LYS(924)	+ 28.15	–36.13
D23	^1100^ARG(933)		^1029^LYS(933)	+ 3.63	–
D24	^1141^LYS(974)		^1073^ARG(977)	+ 32.15	+ 90.85

**Table 2:** Biomolecule binding locations, which overlap with the favourable DOGDIC cross-linking sites.

Cross-link	Aligned ECM binding sites	Enthalpy (kcal/mol)
4	Heat Shock Protein 47 [22], Interleukin-2 [23], α2β1 integrin [24]	–8.68
11	Heat Shock Protein 47 [22], Phosphophoryn [25], Keratan Sulfate PG [9], [21]	–14.33
18	Heat Shock Protein 47 [22], α2β1 integrin [24], Matrix Metalloproteinase 1 [26]	–20.38
19	α2β1 integrin [24], Heat Shock Protein 47 [22], Amyloid Precursor Protein, Interleukin-2 [23], Dermatan Sulfate [27]	–61.58
20	Dermatan Sulfate [27], Interleukin-2 [23], Amyloid Precursor Protein [21]	–4.85
21	Interleukin-2 [23], heparin [28], Amyloid precursor protein [21]	–1.62
